# A Phylogenomic Study of Human, Dog, and Mouse

**DOI:** 10.1371/journal.pcbi.0030002

**Published:** 2007-01-05

**Authors:** Gina Cannarozzi, Adrian Schneider, Gaston Gonnet

**Affiliations:** Institute of Computational Science, ETH Zurich, Zurich, Switzerland; University of California San Diego, United States of America

## Abstract

In recent years the phylogenetic relationship of mammalian orders has been addressed in a number of molecular studies. These analyses have frequently yielded inconsistent results with respect to some basal ordinal relationships. For example, the relative placement of primates, rodents, and carnivores has differed in various studies. Here, we attempt to resolve this phylogenetic problem by using data from completely sequenced nuclear genomes to base the analyses on the largest possible amount of data. To minimize the risk of reconstruction artifacts, the trees were reconstructed under different criteria—distance, parsimony, and likelihood. For the distance trees, distance metrics that measure independent phenomena (amino acid replacement, synonymous substitution, and gene reordering) were used, as it is highly improbable that all of the trees would be affected the same way by any reconstruction artifact. In contradiction to the currently favored classification, our results based on full-genome analysis of the phylogenetic relationship between human, dog, and mouse yielded overwhelming support for a primate–carnivore clade with the exclusion of rodents.

## Author Summary

Some basal relationships in the eutherian tree have been difficult to resolve, probably because the underlying divergences took place within a very short period of time. In this study we examine particularly the relationship between human (primates), dog (carnivores), and mouse (rodents). Previous morphological and molecular studies using different datasets and reconstruction methods have come to different conclusions about the relative placement of these orders on the mammalian tree. Here, we use completely sequenced nuclear genomes and a number of different phylogenetic methods to address this difficult problem. An approach of this kind has only recently become possible with the sequencing of several complete mammalian genomes including the opossum as a relevant outgroup. Our results strongly suggest a sister relationship between primates and carnivores.

## Introduction

A correct interpretation of the direction of evolution in basal parts of the mammalian tree has important implications for different aspects of biology and also for medicine (e.g., the selection of appropriate model organisms). However, some basal relationships may still need further examination before being considered as conclusively and finally settled. Paleontological data show a sudden radiation of mammals in the late Cretaceous [1]. Molecular data might resolve the succession of the early diversification events of placental mammals, but molecular analyses in general suggest an earlier timeframe [2,3]. In particular, the phylogenetic positions of rodents, primates, and carnivores are still contentious, with traditional morphology supporting a primate–rodent clade [4] (called Supraprimates or Euarchontoglires) and molecular studies showing support for either a primate–rodent clade [5–9] or a primate–carnivore clade [10–12]. The results of Jorgensen et al. [13] support a rodent outgroup to a primate–artiodactyl clade based on full genome analyses. Lin et al. [14] report a primate–rodent clade but only after constraining the rodents to be strictly monophyletic. Mitogenomic studies almost invariably support the primate–carnivore clade including that of Janke et al. [15], who presented the first marsupial rooting of the eutherian tree. This topology has also been confirmed in subsequent studies using mixed data [16,17]. The molecular studies differed in the type (nuclear, mitochondrial, or both) and in the amount of genomic data (more species versus more genes) as well as in the tree reconstruction methods.

The inconsistency among these results underlines the difficulty in resolving the three-taxon relationship involving rodents, primates, and carnivores. The short branches separating these groups reside deep within the mammalian phylogenetic tree, thereby enhancing the effects of any reconstruction artifacts. These can be related to data quality or any failure to accurately model particular aspects of evolution such as parallel evolution, lineage specific mutation rates, or other changes in the evolutionary process [14].

Long branch attraction (LBA) may occur when an ingroup has a faster rate of evolution, thereby promoting migration of the long branch with accelerated evolution toward the long branch of the outgroup. This phenomenon was first examined by Felsenstein [18], who showed that trees with long branches could be positively misleading when reconstructed under the parsimony criterium. Parsimony, which computes the minimum number of evolutionary steps required to explain the observed sequences, however, does not have the properties of distance and is not additive. Additive means that for a lineage *A* → *B* → C, the equation *d_AB_* + *d_BC_* = *d_AC_* is satisfied in the expected value. In case any particular taxon (e.g., mouse [19]) evolves faster than the other two in our three-taxon analysis, LBA could possibly affect the outcome for parsimony or nonadditive distance measures. Additive distance estimates such as those produced by the Markov model of evolution used in this study should not be affected by LBA. However, systematic biases such as those produced by parallel evolution or other deviations from the model can affect any evolutionary distance measure.

Parallel morphological or molecular evolution can occur when two species develop similar characteristics because of adaptation to similar environments or life strategies. A coupling between molecular and morphological evolution among mammals is highly speculative, however. To counter any systematic biases, we have made the precaution of using different phylogenetic methods based on different evolutionary phenomena because it is unlikely that all methods will be affected by systematic biases in the same way.

Taxon sampling may affect the accuracy of phylogenetic reconstruction [20–23]. Some authors argue that increasing the number of characters sampled per taxon improves the accuracy, while others state that accuracy is better improved by subdividing long branches by including more taxa, resulting in fewer characters overall. In any case, the choice of more characters versus more taxa depends on the phylogeny under consideration. In the problem under investigation here, a very short branch separates two possible phylogenies, and the comparison is between the number of mutations that occurred on the short common branch and the number of homoplasies that occur on the longer branches. With increased character sampling, we increase the chance of detecting the relatively few changes that occur on the common branch. In certain cases, increasing the number of taxa is useful to divide long branches to help to identify homoplasies [24]. Therefore, in an extended analysis, we also included all available mammalian genomes.

To combat the problems inherent in elucidating this difficult topology, we used a wide range of methods that measure different aspects of molecular evolution, with the view that it is very unlikely that a specific change in the evolutionary process (e.g., different DNA repair mechanisms in murid rodents [14]) would affect *all* of the measures used in this study.

## Results/Discussion

### Phylogenetic Analysis

The evolutionary relationship of dog (*Canis familiaris,* order Carnivora), mouse (*Mus musculus,* order Rodentia), and human (*Homo sapiens,* order Primates) was examined applying a wide variety of distance measures and using the opossum (Monodelphis domestica) as the outgroup because, as a marsupial, it is the closest relative to the eutherian dataset. Thus, solving the problem of the phylogeny of the mouse, human, and dog requires finding the root of the tree. This can be achieved by placing the outgroup on one of the three branches leading to the orders. Figure 1 shows the three possible positions of the outgroup as well as the resulting rooted trees for the opossum and the three eutheria. We endeavored to answer the question of which of these three hypotheses—a primate–carnivore clade, a primate–rodent clade, or a rodent–carnivore clade—represents the true phylogeny.

**Figure 1:**
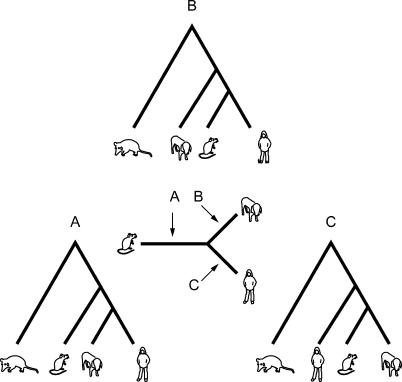
Rooted and Unrooted Topologies Unrooted trees of three species (human, dog, mouse) display no information about the speciation order (center triplet). Only the use of an outgroup (opossum) places the root on one of the three branches (labeled A, B, and C), giving three possible rooted trees corresponding to the three hypotheses being tested.

#### Measures of genomic distance.

To counteract the possible influence of reconstruction artifacts, we applied a collection of methods for tree reconstruction and included a collection of additional complete mammalian genomes. Distance trees were constructed based on a variety of genomic distance measures, parsimony trees were evaluated with and without gapped positions, and likelihood trees were computed on multiple sequence alignment (MSA) columns containing no gaps.

The three types of genomic distance measures used—amino acid replacement, synonymous nucleotide substitutions, and gene reordering—measure different aspects of molecular evolution. All measures correlate with time but are measuring independent properties in that a distance of 0 in one measure does not necessarily affect the distance computed from another measure. For example, synonymous substitutions are independent of amino acid replacement while both are independent from gene reordering. Table 1 and the following subsections summarize and discuss the results from the various methods used.

**Table 1:**
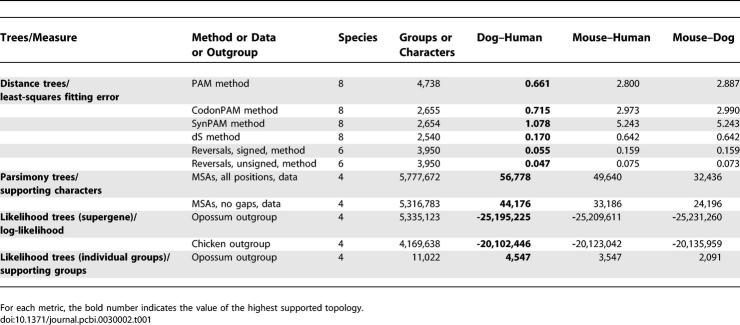
Summary of the Methods Used and Their Results

#### Distance trees built using mean PAM, CodonPAM, and SynPAM between orthologs.

Four methods were employed to measure the evolutionary change between two proteins or their coding DNA sequence: PAM, CodonPAM, SynPAM, and dS. PAM distance is a long-used measure of evolutionary distance of protein sequences, which estimates the distance using empirically determined amino acid mutation matrices [25]. SynPAM [26] and CodonPAM [27] are recent extensions of the empirical model to coding DNA. Both methods are based on a 64 × 64 mutation matrix describing substitution probabilities between any two codons. The SynPAM method estimates the distance only on positions with conserved amino acids, while CodonPAM uses all aligned codons. Therefore, CodonPAM combines the synonymous substitutions with amino acid replacing changes and has been shown to improve the accuracy of distance estimates [28].

Synonymous mutations in coding DNA do not alter the encoded protein. Thus, they are under no strong selection pressure and are less constrained by functional changes. Because of these properties they are particularly robust against the effects of parallel evolution. Therefore we employed a second method measuring synonymous changes, the number of synonymous substitutions per synonymous site, dS.

Distance trees using PAM, CodonPAM, SynPAM, and dS distances were created from the complete set of orthologous groups from eight mammals with completely sequenced genomes. Adding more in-group genomes reduces the number of complete groups of orthologous sequences, but adds more intergenome distances, making the trees more robust and reducing possible biases from particular genomes. All of the trees constructed using any of these four distance measures supported the primate–carnivore clade as shown in Figure 2A. To assess the reliability of the resulting trees, bootstrapping was performed by sampling orthologous groups. Bootstrapping is an empirical way of estimating the variance of a result without knowledge of the underlying distribution. Using very large amounts of data, as in this study, leads to a very low variance, and therefore the results from the bootstrapping are always 100%. For this reason, they are not reported in Figure 2. The fit of the distances to each of the three topologies in Figure 1 was computed for each method via least squares, and the residuals (normalized by degrees of freedom) are reported in Table 1.

**Figure 2:**
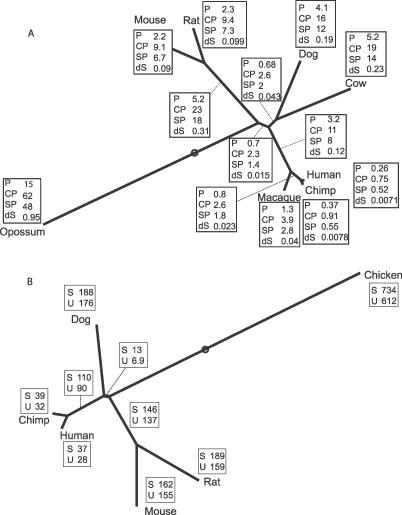
Distance Phylogenetic Trees of Mammals (A) The phylogenetic tree of the mammals is shown here with branch lengths obtained using different distance criteria shown in the tables: PAM distance (P), CodonPAM (CP), SynPAM (SP), and dS (dS). The branch lengths shown are proportional to PAM distance, and the circle indicates the placement of the root. (B) The phylogenetic tree of the mammals constructed using signed (S) and unsigned (U) reversal distance, with branch lengths proportional to the number of signed reversals.

#### Distance trees obtained using reversal distances.

Several mechanisms can alter the ordering of genes on and across chromosomes. As changes of this kind accumulate over time and eventually become fixed in a population, the number of such changes between two genomes can serve as an evolutionary distance. One of the simplest operations to model genome reordering is an inversion or reversal, where a part of the DNA is removed from the strand and reinserted in the reverse direction. The minimal number of such inversion operations that are needed to transform the gene order in one genome to the order in another genome is called the reversal distance. Both signed and unsigned reversal distance (if the direction of the gene is considered or not) were used to compute distance trees for human, chimpanzee, dog, mouse, rat, and chicken. The percentage of inversions observed for the most distant pair (chicken versus rat), were 27% for the signed and 23% for the unsigned versions of the algorithm. Tests on simulated data revealed that for these distances the exact number of reversals was found more than 99% of the time (unpublished data). This means that for this distance range, multiple inversions almost never occur in a way that could be explained by fewer reversals, which would cause an underestimation of the distance. The tree obtained by this method, again supporting a rodent outgroup to primates/carnivores, is shown in Figure 2B. The normalized residuals from the least squares fitting for each topology are given in Table 1.

### Parsimony Analysis of Characters from Multiple Sequence Alignments

MSAs for parsimony analysis were created for the three eutheria plus opossum with aligned amino acid positions considered as characters. The numbers of positions supporting each topology were counted using either all informative positions or excluding positions with gaps and are summarized in Table 1. Because sequence evolution is a stochastic process and the branch separating the conflicting hypotheses is very short, the absolute difference between the numbers of characters supporting each hypothesis can be small. However, with the large number of characters available, the variance of the estimation is small compared with the large numbers of supporting positions. In Table 2 the significance of the number of positions supporting the primate–carnivore clade compared with the primate–rodent clade are reported in standard deviations and are very significant.

**Table 2:**
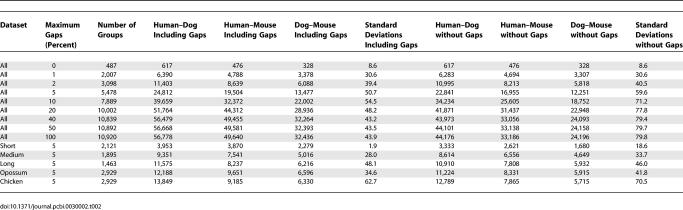
Number of MSA Positions Supporting the Three Topologies under the Parsimony Criterium and the Significance of the Difference as a Function of the Percentage of Allowable Gaps

Some genes from the complete genomes are only fragments, and this may result in the MSAs being of reduced quality. Therefore, in a second analysis, alignments with a high frequency of gapped positions were excluded. The results of analysis with and without gaps are shown in Table 2, in which the numbers of characters supporting each topology are shown as a function of the allowable percentage of gapped positions in the alignment. It is interesting that for the analysis including gapped positions (columns 4–7 in Table 2), as the percentage of gaps allowed in the groups increases from 1% to 10%, the support for the mouse outgroup hypothesis becomes stronger because of the increase in data occurring with the addition of groups. As the allowed percentage of gaps exceeds 10%, the quality of the data deteriorates and the significance of the support for the mouse outgroup hypothesis decreases. When all positions containing gaps are excluded (columns 8–11 in Table 2), the support for the primate–carnivore clade continually increases with increasing amounts of data.

In an extended analysis, the groups of genes were broken into thirds—short, medium, and fast-evolving—based on the sum of all pairwise distances. The results for those with ≤5% gapped positions are reported in Table 2. Each of the thirds supports the same primate–carnivore grouping. The decrease in significance with decreasing evolutionary distance can be attributed to the decreasing amount of informative positions as the sequences become more similar.

To assess the influence of the choice of the outgroup, all genes represented by the three eutheria and the chicken and the opossum and containing at most 5% gaps were analyzed. Both outgroups support a primate–carnivore clade, although the significance decreases when the opossum is used as the outgroup.

### Likelihood Analysis

The same MSAs as for the parsimony analyses were used for a likelihood analysis of quartets using either the chicken or the opossum as an outgroup. First, all genes in the orthologous groups were concatenated to form one supergene. The log-likelihoods of the data given each of the three topologies in Figure 1 were computed and are shown in Table 1. For both outgroups, the likelihood is orders of magnitude greater for the topology supporting a primate–carnivore clade than for the alternatives. Because different orthologous groups were included for the analysis of each outgroup, the likelihoods between the outgroups are not comparable. A second analysis was performed by taking all orthologous groups containing the opossum, creating a gene tree for the four sequences in each group, and computing the likelihood of the data for each topology. The number of gene trees supporting each topology was counted, resulting in a clear majority supporting the primate–carnivore clade.

### Conclusions

The analysis of the three-taxon relationship (mouse/human/dog) based on data from complete nuclear genomes strongly suggested a sister relationship between human (primates) and dog (carnivores) to the exclusion of mouse (rodents). The limited length of the branch separating the topologies may make analysis of the tree sensitive to the choice of evolutionary model and data. Therefore, the analyses were conducted applying a number of independent methods to the genome-sized datasets, all of which supported this relationship. The effects of adding more taxa versus sampling more characters were also investigated. Inclusion of additional genomes (rat, chimp, macaque, cow) did not change the topology of the tree. However, sampling many characters yielded very significant support for the short internal branch. Therefore, we suggest that this difficult phylogenetic problem can only be solved using thousands of genes, which are only available from complete genomes. When the critical branch is so small, the use of a large number of genes is the only way to reduce the variance enough to get statistically significant results.

## Materials and Methods

### Data and implementation.

All analyses were performed on fully sequenced genomes from human, dog, mouse, and opossum. As an extension, up to four other mammalian genomes were included in some analyses as was the complete genome of the chicken, which was used as an alternative outgroup. The genomes downloaded from ENSEMBL [29] had version numbers NCBI 35 for human [30], BROADD1 for dog, NCBI m36 for mouse [19] and 0.5 for opossum. The other genome databases (Bos taurus (Btau_2.0), Gallus gallus (WASHUC1), Pan troglodytes (CHIMP1), Rattus norvegicus (RGSC3.4), and Macaca mulatta (Mmul 0.1) ) are also from ENSEMBL. All databases were converted to the *Darwin* database format for further computations. The implementation of the methods was entirely done in the *Darwin* programming language [31] with the exception of the computation of dS.

### Orthologs.

Groups of orthologous proteins from the orthologous matrix (OMA) project [32] constituted the basis for building the trees. The OMA project is a large-scale effort to identify groups of orthologous sequences in a fully automated manner. This is achieved by computing all-against-all sequence alignments between all sequenced genomes from all kingdoms of life (297 genomes completed at the time of writing). The OMA groups are conservative in that a careful search for possible paralogy discards all suspicious matches. Every candidate pair of sequences is verified against all other genomes to identify gene loss that could lead to inclusion of paralogs. The orthology assignments are done without assuming an underlying species tree, which would cause a bias for the inference of a phylogeny. In the latest OMA release, we find 11,022 groups having a representative in each of the four primary species under study.

### Phylogenetic methods.

Distance trees were calculated using the PhylogeneticTree package in *Darwin*. For distance trees, all pairwise distances and variances are estimated, and a tree is sought that best approximates the distance information via weighted least squares. Finding the best-fitting tree is an NP-complete problem [33]. The polyalgorithm implemented in *Darwin* solves the problem in the following way: one neighbor-joining tree and 29 trees with random topologies are created as starting points. All trees are then optimized using branch-swapping heuristics, and the best-fitting tree is retained. When considering only four or five species, the exact computation of the best tree could be done manually (three or 15 topologies to analyze, respectively). For relatively small problems such as those encountered here (at most eight leaves), the algorithm almost certainly finds the optimal topology. This was verified by simulation studies (unpublished data).

### Evolutionary distance.

The PAM, CodonPAM, and SynPAM methods were implemented in *Darwin*. Proteins and coding sequences were aligned using dynamic programming [34,35], and the distances were estimated by maximum likelihood. The likelihood method implemented in the *codeml* program from the *PAML* software package [36] was used to compute dS from pairwise alignments of coding DNA.

Only complete groups of orthologs (groups with one member in all genomes under consideration) were used to compute the average distances between two species. The distances from all pairwise alignments of orthologous sequences are averaged for each pair of species, resulting in a distance and a variance matrix from which trees are built. For DNA-based methods, groups had to be excluded when the coding DNA for at least one of the member proteins was not or was only partially available. Also excluded were alignments with fewer than 100 synonymous positions because distance estimates based on short alignments suffer from statistical biases. Additionally, groups were excluded from the SynPAM analysis if at least one distance estimate was higher than 1,000 (corresponding to approximately ten substitutions per synonymous codon). For the dS analysis, groups with one value higher than ten were discarded. Such high values indicate that the synonymous substitutions between two genes are saturated and thus have a very high variance.

### Gene reordering (reversal distance).

The gene orders of two genomes can be formulated as a permutation of a list of integers, referring to the order of orthologous genes in the genomes. As an example, we consider two genomes A and B with only three genes, where the first gene in A is orthologous to the second gene in B, and vice versa, while the third genes in both genomes are orthologous. Stated as a permutation, the genes [1, 2, 3] in A are transformed to [2, 1, 3] in B. A reversal operation inverts a subsequence of the gene order. Applying this operation to the first two genes will transform the gene order in A to the one in B. Therefore, the reversal distance between A and B is one. If the direction of the genes is known and used for the computation, this is called “signed” (because the numbers in the permutations are labeled with a minus sign if the genes are found on the complement strand of the DNA) and can be computed in linear time [37,38]. If the direction of the gene is not known, it is called “unsigned.” In this case, the problem of finding the optimal sequence of reversals is NP-hard [39]. We implemented a k-greedy algorithm in *Darwin* to solve the unsigned problem.

Computing the reversal distance can only be done reliably if large stretches of the genome are assembled. Unfortunately, our version of the opossum genome was only assembled into scaffolds, but not yet into complete chromosomes. Therefore, we used the chicken as the outgroup because the genes are already placed on chromosomes. For the same reason, macaque and cow could not be used for this method. Because only a very small number of reversals decide the phylogeny in this study, we filtered the orthologous groups as much as possible to reduce noise. Groups were excluded from the analysis if at least one gene was placed on a scaffold instead of a chromosome or if two genes had overlapping coding regions.

### Parsimony over multiple-sequence alignments.

MSAs of orthologs from four species—human, mouse, dog, and opossum—were built using two methods available in *Darwin,* probabilistic and circular tours [40]. In *Darwin,* MSAs can be improved using gap-rearrangement heuristics. All the MSAs are scored and the best-scoring one selected. 102 alignments were eliminated from the analysis for having fewer than 100 positions, leaving 10,920 groups. To eliminate the influence of gene fragments and misfound start and stop positions, the alignments were truncated at both ends—only characters between the first and last position containing no gap in any sequence were counted. To decide which of the three quartets is the most parsimonious one, only those alignment positions at which two species share one character and the other two share another character (2–2 cases) are informative. (Positions where all species share the same characters or have all four different, as well as 3–1 splits, are uninformative. The 2-1-1 splits have parsimony costs of 2 for all three topologies and are also uninformative. Thus, only the characters that have a 2–2 split are of interest.) The standard deviations separating the two topologies in Table 2 are computed under the assumption of a binomial distribution of the counts for the primate–carnivore clade (*n*_1_) and the primate–rodent clade (*n*_2_ **)** as:

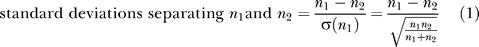



### Likelihood analysis.

Likelihood trees were also implemented in *Darwin*. Since optimizing topology and branch lengths of likelihood trees is very time-consuming, only trees with four leaves (human, mouse, dog, and one of opossum and chicken) were computed. Likelihood trees were constructed for each group (MSA) individually and for the concatenated alignments. Positions containing a gap in any of the four sequences were completely ignored. Optimizing the lengths of the five branches for a given quartet topology was initialized with the branch length of the least squares tree, and then numerically improved first by steepest-ascent, and then by multidimensional Newton. The optimization of the likelihood for one topology over approximately 5 million characters was computed in about one hour on a desktop Linux machine.

## Abbreviations

LBA: long branch attraction
MSA: multiple sequence alignment
OMA: orthologous matrix
